# Endothelial STING controls T cell transmigration in an IFNI-dependent manner

**DOI:** 10.1172/jci.insight.149346

**Published:** 2021-08-09

**Authors:** Marina Anastasiou, Gail A. Newton, Kuljeet Kaur, Francisco J. Carrillo-Salinas, Sasha A. Smolgovsky, Abraham L. Bayer, Vladimir Ilyukha, Shruti Sharma, Alexander Poltorak, Francis W. Luscinskas, Pilar Alcaide

**Affiliations:** 1 Department of Immunology, Tufts University School of Medicine, Boston, Massachusetts, USA.; 2 Department of Internal Medicine, University of Crete Medical School, Crete, Greece.; 3 Center for Excellence in Vascular Biology, Department of Pathology, Brigham and Women’s Hospital, Boston, Massachusetts, USA.; 4 Harvard Medical School, Boston, Massachusetts, USA.; 5 Tufts Graduate School for Biomedical Sciences Immunology Program, Tufts University School of Medicine, Boston, Massachusetts, USA.; 6 Petrozavodsk State University, Petrozavodsk, Republic of Karelia, Russia.

**Keywords:** Inflammation, Vascular Biology, Cell migration/adhesion, Endothelial cells

## Abstract

The stimulator of IFN genes (STING) protein senses cyclic dinucleotides released in response to double-stranded DNA and functions as an adaptor molecule for type I IFN (IFNI) signaling by activating IFNI-stimulated genes (ISG). We found impaired T cell infiltration into the peritoneum in response to TNF-**α** in global and EC-specific STING^–/–^ mice and discovered that T cell transendothelial migration (TEM) across mouse and human endothelial cells (EC) deficient in STING was strikingly reduced compared with control EC, whereas T cell adhesion was not impaired. STING^–/–^ T cells showed no defect in TEM or adhesion to EC, or immobilized endothelial cell–expressed molecules ICAM1 and VCAM1, compared with WT T cells. Mechanistically, CXCL10, an ISG and a chemoattractant for T cells, was dramatically reduced in TNF-**α**–stimulated STING^–/–^ EC, and genetic loss or pharmacologic antagonisms of IFNI receptor (IFNAR) pathway reduced T cell TEM. Our data demonstrate a central role for EC-STING during T cell TEM that is dependent on the ISG CXCL10 and on IFNI/IFNAR signaling.

## Graphical abstract

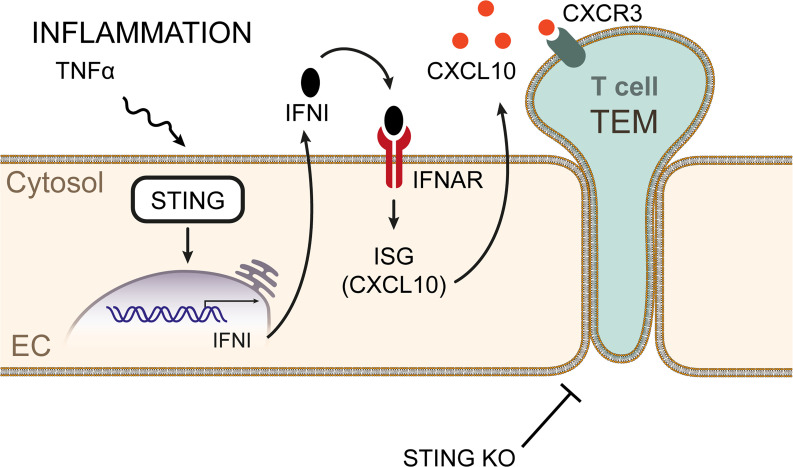

## Introduction

Mounting an inflammatory response requires a highly regulated multistep process that involves adhesive interactions of the leukocytes with the vascular endothelium. Endothelial selectins and integrin ligands adhere to leukocyte-expressed selectin ligands and integrins, and the receipt of chemoattracting signals promotes leukocyte firm arrest and transendothelial migration (TEM) (1).

Stimulator of IFN genes (STING) is an endoplasmic reticulum protein that contributes to innate immune responses triggered by cytosolic dsDNA (2). Double-stranded DNA (dsDNA) activates cytoplasmic DNA sensors that generate cGAMP that directly bind to STING and induce its phosphorylation and translocation to the Golgi-endoplasmic reticulum (endoplasmic-reticulum–Golgi intermediate compartment [ERGIC]). STING associates with TBK-1, IRF3, activated NF-κB and other factors (3) to form a multimolecular “signalosome” complex that promotes the transcription of IFN-stimulating genes (ISG), such as CXCL10, a chemoattractant for Th1 cells (4–6), and ΝF-κΒ–activated genes (7–10). In humans, gain-of-function mutations in *TMEM173*, the gene that encodes for STING, result in STING-associated vasculopathy with onset in infancy (SAVI), an inflammatory disease characterized by upregulated expression of genes associated with the type I IFN (IFNI) pathway (11). Studies in global *Tmem173^–/–^* mice (STING^–/–^ mice) support that STING promotes inflammation in many experimental models (12–15), whereas it is critical to maintaining homeostasis and preventing inflammation in other models (16, 17). In endothelial cells (EC), STING has been primarily studied as an inducer of IFNI responses following viral infections (18). A recent study has demonstrated that STING activation in EC occurred in a model of sterile inflammation induced by free fatty acids (12). However, the cell-specific role of STING in these various inflammatory settings is poorly understood. Specifically, the role of STING in leukocyte TEM and whether the cell-specific intrinsic actions of STING are mediated through IFNI or NF-κB activation remain to be investigated. Here, we tested the hypothesis that EC-STING is required for leukocyte TEM. We report a central role for EC-STING in T cell recruitment and TEM in response to TNF-α in vitro, and in vivo, that is dependent on the ISG CXCL10 and IFN-type I IFN receptor (IFNAR) signaling.

## Results

### Impaired leukocyte recruitment in response to TNF-α and thioglycollate-induced peritonitis in STING^–/–^ mice compared with WT mice.

**Figure 1:**
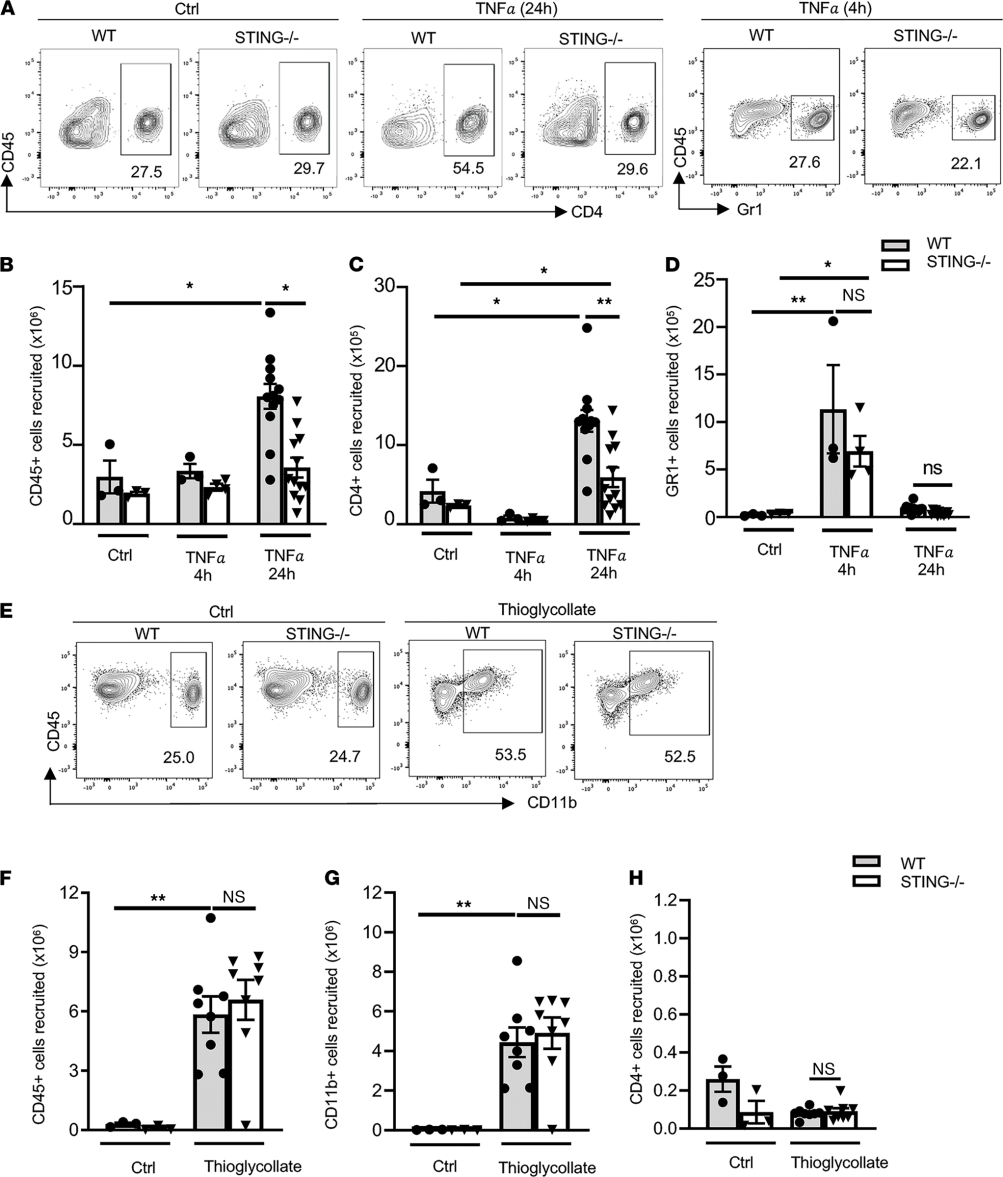
Impaired leukocyte recruitment in response to TNF-α–induced peritonitis in STING–/– mice compared with WT mice. (**A**–**H**) WT and STING^–/–^ mice received PBS, TNF-α 4 hours (h), or TNF-α 24 h (**A**–**D**) or thioglycollate 3 days (**E**–**H**) via i.p. injection, and the peritoneal lavage was analyzed by FACS. (**A**) Representative flow cytometric panels of CD45^+^CD4^+^ cells recruited to the peritoneal cavity at 24 h and of CD45^+^Gr1^+^ at 4 h. (**B**–**D**) Quantification of total CD45^+^ (**B**), CD45^+^CD4^+^ (**C**), and CD45^+^Gr1^+^ (**D**) recruited to the peritoneal cavity. *n* = 3 (control); *n* = 12 (TNF-α 24 h); *n* = 3-4 (TNF-α 4 h). (**E**) Representative flow cytometric panels of CD45^+^CD11b^+^ cells recruited to the peritoneal cavity in response to thioglycolate of 3 independent experiments. (**F**–**H**) Quantification of CD45^+^ (**F**), CD45^+^CD11b^+^ (**G**), and CD45^+^CD4^+^ (**H**) recruited cells to the peritoneal cavity. *n* = 3 (control); *n* = 8 (thioglycollate). Data are shown as mean ± SEM values. *P < 0.05 and **P < 0.01; 2-way ANOVA (**B**–**D**) and 1-way ANOVA (**F**–**H**).****

We used WT and global STING-deficient mice in 2 acute models of sterile inflammation to test whether STING contributes to immune cell recruitment. We first used a well-established model of peritonitis induced by TNF-α to assess CD4^+^ T cell and Gr1^+^ neutrophil recruitment (19, 20). After 24 hours of TNF-α stimulation, we observed a 3-fold increase in the recruitment of CD45^+^ immune cells into the peritoneal cavity of WT mice as compared with PBS-treated control mice. In contrast, STING^–/–^ mice exhibited a ~50% decrease in CD45^+^ leukocyte recruitment (Figure 1, A and B). We further quantified CD4^+^ T cells and Gr1^+^ neutrophils within the CD45^+^ leukocyte gate using the gating strategy shown in Figure 1A and Supplemental Figure 1A (supplemental material available online with this article; https://doi.org/10.1172/jci.insight.149346DS1). CD4^+^ T cells were abundantly recruited to the peritoneal cavity at 24 hours after TNF-α treatment in WT mice, whereas a 50% decrease in CD4^+^ T cell recruitment was observed in STING^–/–^ mice (Figure 1C). Gr1^+^ neutrophil recruitment was very low, and there was no significant defect in STING^–/–^ compared with WT mice at 24 hours (Figure 1D). Because the recruitment of neutrophils was 10-fold less than that of CD4^+^ T cells at the 24-hour time point, we performed additional studies at 4 hours after TNF-α, which coincides with robust neutrophil recruitment, as we and others have previously reported (20). As expected, neutrophil infiltration into the peritoneum was evident at 4 hours and significantly declined at 24 hours, with no significant difference between WT and STING^–/–^ mice (Figure 1, A and D). In contrast, the CD4^+^ T cell infiltration, which was robust at 24 hours, was minimal at 4 hours, with no difference in recruitment between WT and STING^–/–^ mice (Figure 1C). 

We further investigated if STING was required for thioglycollate-induced leukocyte recruitment, a well-established model of sterile inflammation that predominantly induces recruitment of monocytes and macrophages 3 days after injection (Supplemental Figure 1B) (21). As expected, thioglycollate induced significant recruitment of CD45^+^ cells in WT mice (Figure 1, E and F), and within the CD45^+^ gate, the majority of cells were CD11b^+^ myeloid cells (Figure 1G). Interestingly, we observed similar numbers of CD45^+^CD11b^+^ and CD4^+^ T cells in the peritoneal cavity of STING^–/–^ mice treated with thioglycollate (Figure 1, E–H). Taken together, our data indicate that leukocyte recruitment in STING^–/–^ mice is impaired in response to TNF-α but not in response to thioglycollate challenge. Our results suggest that STING predominantly regulates CD4^+^ T cell recruitment in response to TNF-α.

### STING deficiency in EC but not in T cells results in impaired T cell TEM in response to TNF-α.

**Figure 2:**
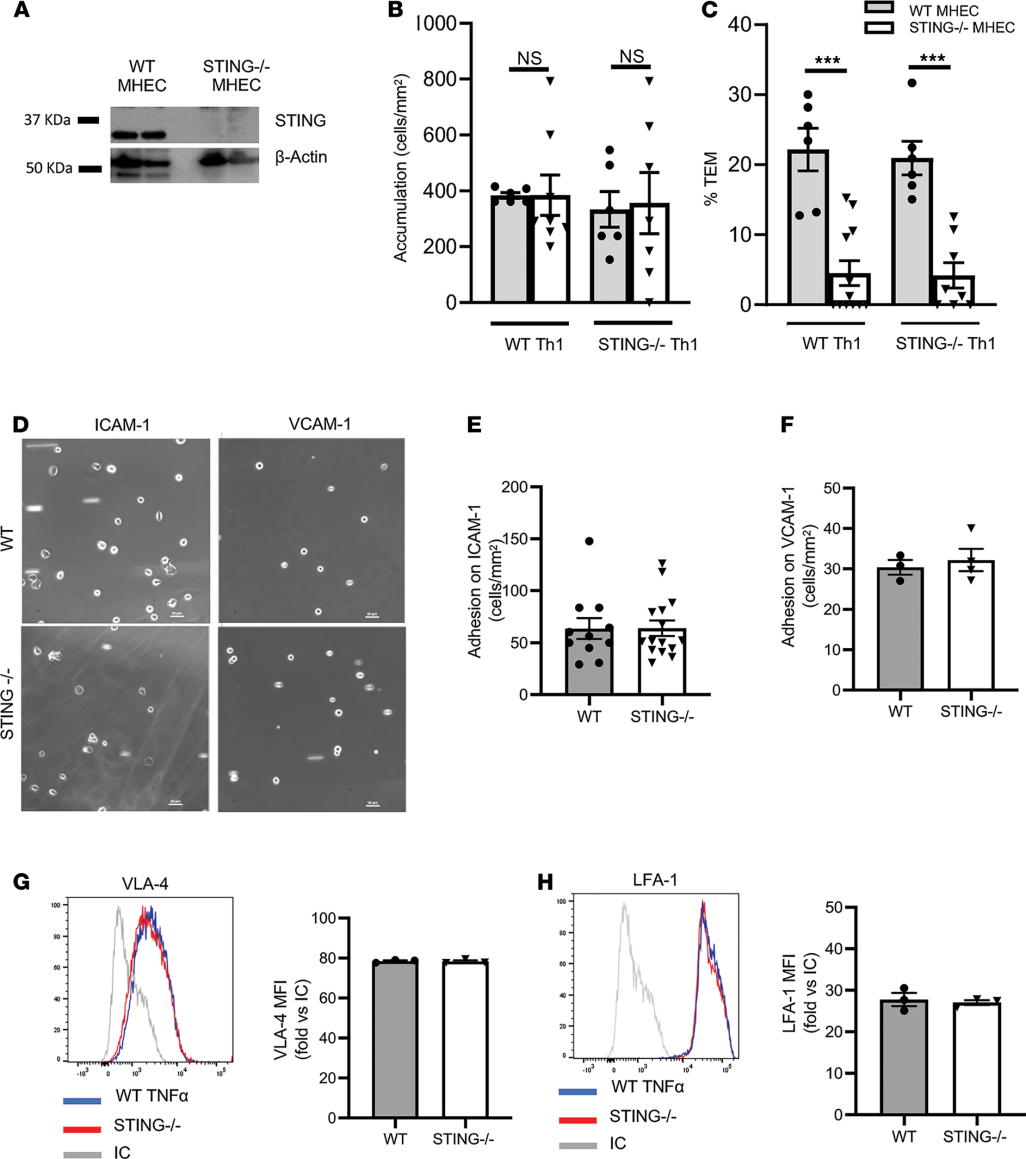
STING deficiency in EC but not in T cells results in impaired TEM in response to TNF-α. (**A**) Cultured MHEC from WT and STING^–/–^ mice were lysed and analyzed by immunoblot to evaluate STING expression and β-actin, used as a loading control. (**B **and** C**) Quantification of adhesion and %TEM of WT and STING^–/–^ Th1 cells perfused across WT and STING^–/–^ MHEC (for WT Th1 groups: *n* = 3 independent experiments with WT and STING^–/–^ MHEC preparations and Th1 preparations, using duplicate or triplicate coverslips). (**D**) Representative images of WT and STING^–/–^ Th1 cell adhesion on ICAM1- and VCAM1-coated coverslips following perfusion under flow conditions. Scale bar: 100 μm. (**E** and **F**) Quantification of Th1 adhesion on ICAM1 (*n* = 3 independent experiments, triplicate coverslips) and on VCAM1 (*n* = 3 independent experiments). (**G** and **H**) Representative flow cytometry histograms and quantification of VLA-4 (**G**) and of LFA-1 (**H**) from WT and STING^–/–^ Th1 cells (*n* = 3 independent Th1 cell preparations). Data are shown as mean ± SEM. ***P < 0.001; 1-way ANOVA (**B **and** C**) and *t* test (**E **and** H**).

We next investigated whether the impaired T cell recruitment observed in the TNF-α–induced peritonitis in STING^–/–^ mice was mediated by STING expressed in mouse heart EC (MHEC) or in T cells, or in both cell types. We used a well-established in vitro model of TEM under flow conditions that involves primary MHEC and Th1 cells (22, 23) from both WT and STING^–/–^ mice. STING protein was expressed in WT MHEC (Figure 2A), and its expression was similar to that reported for immune cells (24) (Supplemental Figure 2A). As expected, very few WT Th1 cells adhered to unstimulated MHEC, and the few adherent cells did not undergo TEM (Supplemental Figure 2, B and C). We did not observe any differences in the accumulation of Th1 cells to TNF-α–stimulated WT or STING^–/–^ EC (Figure 2B). Strikingly, the percentage of WT Th1 cell TEM induced by TNF-α was significantly decreased in STING^–/–^ MHEC as compared with WT MHEC (Figure 2C). This defect in TEM was not observed when STING^–/–^ Th1 cells were perfused across WT MHEC, and indeed, Th1 cell TEM was significantly impaired only when STING was lacking in MHEC. (Figure 2C). WT and STING^–/–^ Th1 cells exhibited identical levels of intracellular IFN-γ (Supplemental Figure 3), which confirms that STING deficiency does not alter in vitro Th1 cell differentiation. No further defect in TEM was observed when both Th1 cells and MHEC were lacking STING (Figure 2C). WT and STING^–/–^ Th1 cells also showed adhesion comparable with immobilized ICAM1 and VCAM1, which are surface-expressed molecules upregulated in EC by TNF-α activation and are required for leukocyte adhesion and TEM (Figure 2, D–F). As a control, we found that STING^–/–^ and WT Th1 cells expressed similar levels of VLA-4 (Figure 2G) and LFA-1 (Figure 2H), ligands for VCAM1 and ICAM1, respectively. Taken together, these data demonstrate that EC-STING, and not T cell STING, is central to T cell TEM under flow conditions in vitro.

### Specific deletion of STING in EC results in decreased T cell recruitment in response to TNF-α in vivo.

**Figure 3:**
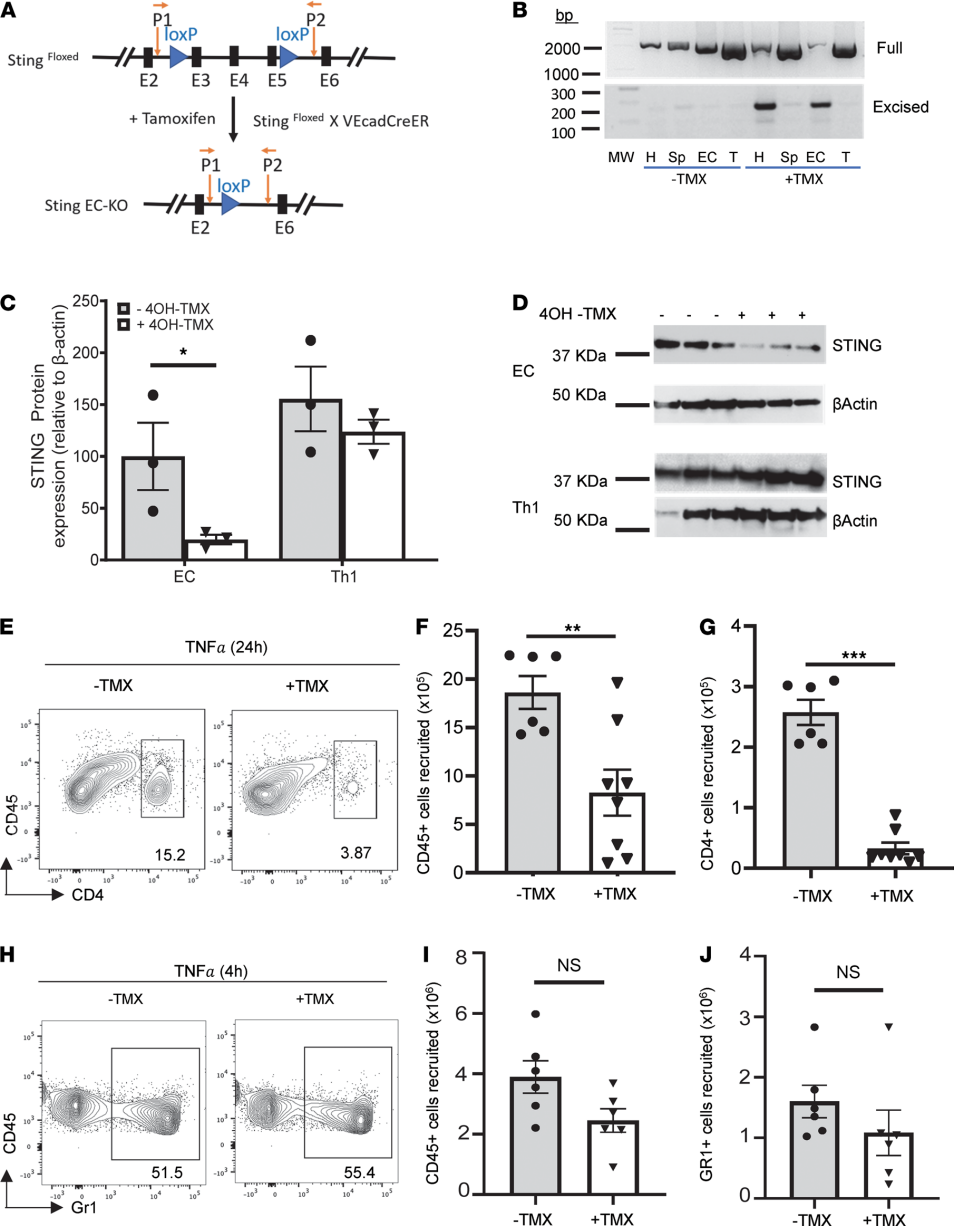
Decreased T cell recruitment into the peritoneal cavity of EC-STING–/– mice in response to TNF-α. (**A**) Schematic gene-targeting map of STING gene showing STING floxed and STING conditional alleles before and after tamoxifen (TMX) treatment (75 mg/kg body weight) and primer (P1 and P2) binding sites, with orange arrows pointing at the LoxP sites. (**B**) Primer pair P1 and P2 were used to detect unexcised and excised STING alleles in the heart (H), Splenocytes (Sp), MHEC (EC), and T cells (T) purified from Cad5^ERTCre2+/–^ STING^fl/fl^ treated with vehicle or TMX. (**C**) Quantification of STING protein expression in cultured MHEC from Cad5^ERTCre2+/–^ STING^fl/fl^ and WT Th1 cells treated with vehicle or 4OH-TMX for 24 h. (**D**) Western blotting images of 4OH-TMX–treated cell lysate. Each line is an independent cell preparation (*n* = 3). (**E**) Representative flow cytometric panels of CD45^+^CD4^+^ cells recruited to the peritoneal cavity 24 h after TNF-α. (**F** and **G**)Quantification of total CD45^+^ (**F**) and CD4^+^ (**G**) cells recruited cells to the peritoneal cavity 24 h after TNF-α. (**H**) Representative flow cytometric panels of CD45^+^Gr1^+^ cells recruited to the peritoneal cavity 4 h after TNF-α. (**I** and **J**) Quantification of total CD45^+^ (**I**) and Gr1^+^ (**J**) cells recruited to the peritoneal cavity 4 h after TNF-α. Data represent *n* = 2 independent experiments; *n* = 3 control and *n* = 4 TMX-treated mice per experiment (24 h TNF-α.); and *n* = 6 animals per group (4 h TNF-α). *P < 0.05, **P < 0.01 and ***P < 0.001; *t* test.

To further investigate the role of EC-STING in T cell recruitment in response to TNF-α in vivo, we generated inducible EC-specific–deficient mice (Figure 3A). To this end, we first validated EC-specific recombination and found no excision band for STING in Cdh5^CreERT2+/–^ STING^fl/fl^ mice in the absence of tamoxifen; an intact band was observed in a vascularized tissue such as the heart, as well as in purified MHEC. In contrast, tamoxifen induced excision of STING in both purified and cultured EC and in the heart, but not in T cells or the splenocytes isolated from tamoxifen-treated Cdh5^CreERT2+/–^ STING^fl/fl^ mice (Figure 3B). Cell-specific deletion of STING was further confirmed (81.3% ± 4.5% reduction) in STING protein expression in MHEC treated with 4OH-TMX in culture. In contrast, STING protein expression was not altered by 4OH-TMX in Th1 cells (Figure 3, C and D). Moreover, when EC-STING^–/–^ mice were challenged with TNF-α i.p., we found a dramatically reduced number of CD45^+^ cells and CD4^+^ T cells in the peritoneal cavity as compared with EC-STING–sufficient mice (Figure 3, E–G) at 24 hours after TNF-α. In contrast, Gr1^+^ neutrophils, which are robustly recruited to the peritoneal cavity 4 hours after TNF-α, were similarly recruited in WT and EC-STING^–/–^ mice at this earlier time point (Figure 3, H–J, and Supplemental Figure 4). Taken together, our data demonstrate that EC-STING is required for CD4^+^ T cell recruitment in response to TNF-α.

### STING modulation of T cell TEM is dependent on the ISG CXCL10.

**Figure 4:**
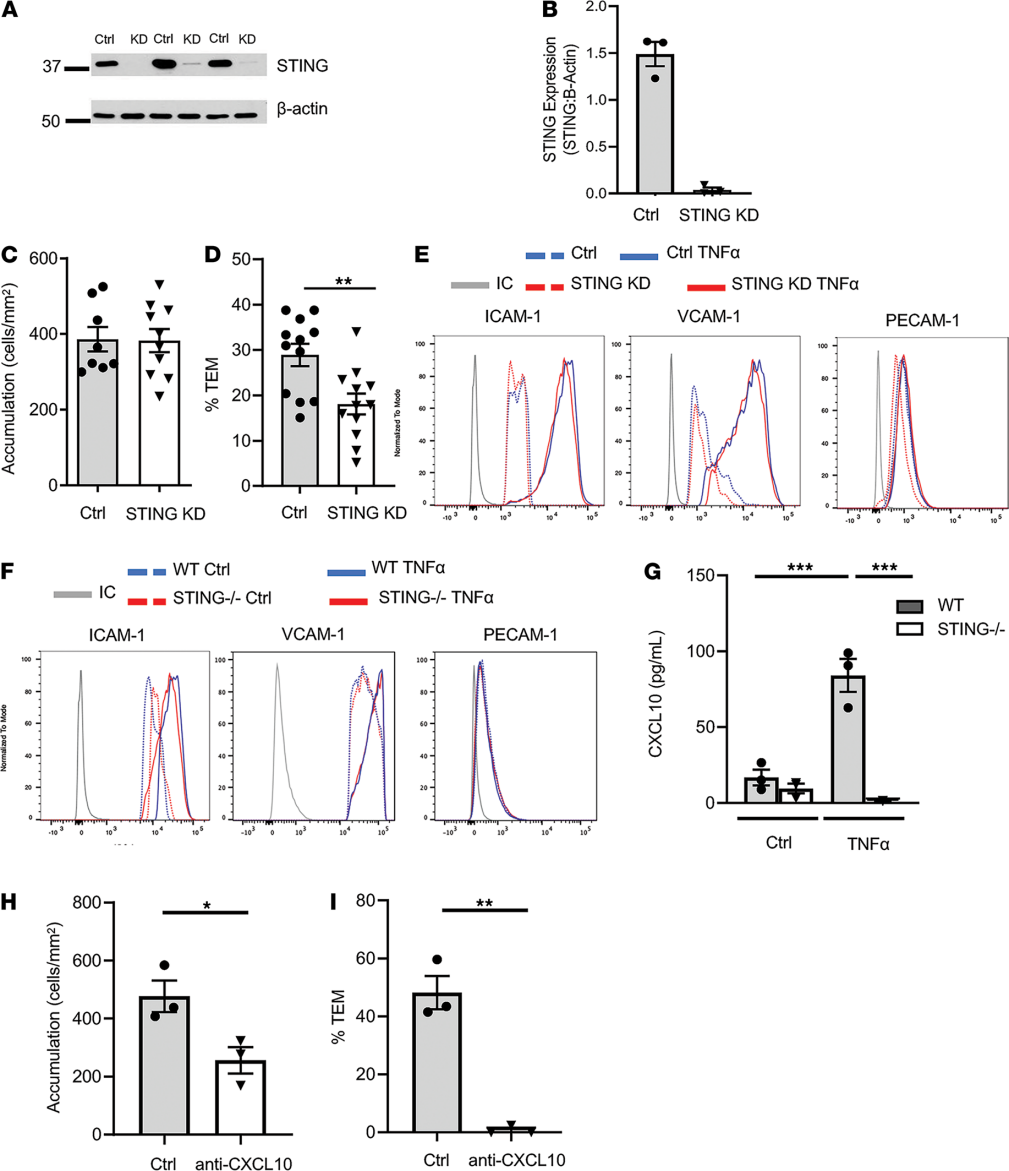
STING modulation of T cell TEM is independent of NF-κΒ–inducible adhesion molecules and dependent on the ISG CXCL10. (**A **and **B**) Immunoblot (**A**) and quantification from 3 independent experiments (**B**) of STING and β-actin expression in control and STING KD HUVEC monolayers using CRISPR. (**C** and **D**) CD3^+^ T cells isolated from human blood were perfused under flow conditions across WT and STING KD HUVEC stimulated for 4 h with TNF-α, and accumulation (**C**) and %TEM (**D**) were quantified. *n* = 8 control and *n* = 10 TNF-α coverslips from 3 independent experiments. (**E **and** F**) Representative histograms of surface PECAM-1, ICAM1, and VCAM1 per cell fluorescent intensity on HUVEC (**E**) and MHEC (**F**). (**G**) Quantification of CXCL10 in supernatants collected from WT and STING^–/–^ MHEC at baseline and in response to 4-h TNF-α stimulation from *n* = 3 cell preparations. (**H **and** I**) Quantification of accumulation (**H**) and %TEM (**I**) of Th1 cells perfused across control and anti-CXCL10-treated MHEC from *n* = 3 independent experiments. Data are shown as mean ± SEM values. *P < 0.05, **P < 0.01 and ***P < 0.001; *t* test.

We further confirmed the role of EC-STING in T cell TEM using CRISPR knockdown of STING (STING KD) in human umbilical vein EC (HUVEC). Using this approach, we achieved 95% KD of STING protein (Figure 4, A and B). Similar to our observations in MHEC, T cell accumulation was comparable between control and STING KD HUVEC monolayers (Figure 4C); however, there was a striking reduction in TEM across STING KD HUVEC, as compared with control (Figure 4D). In contrast, neutrophil TEM was not impaired across STING KD HUVEC as compared with control HUVEC, and similar results were observed with mouse neutrophils across WT or STING^–/–^ MHEC (Supplemental Figure 5). Given the significant reduction in T cell TEM, we hypothesized that STING deficiency led to reduced surface expression of ICAM1 and VCAM1 in EC induced by TNF-α treatment. However, we did not find differences between control and STING KD HUVEC in surface expression of ICAM1 or VCAM1 induced by TNF-α, or in PECAM-1 that was not inducible by TNF-α and is also involved in leukocyte TEM (Figure 4E) (25). Similarly, STING^–/–^ and WT MHEC — which express high baseline levels of ICAM1 and VCAM1, as we have previously reported (22) — had similar expression of ICAM1, VCAM1, and PECAM-1 in response to TNF-α stimulation (Figure 4F).

Th1 cells are characterized by the expression of CXCR3, and recognition of its ligands CXCL9 and CXCL10 is necessary for efficient Th1 cell firm arrest and TEM (26–29). CXCL10 is also an ISG. Thus, we determined the amount of CXCL10 in the conditioned media from control and TNF-α–stimulated WT and STING^–/–^ MHEC, which showed different levels of Th1 cell TEM. As expected, CXCL10 was induced in WT MHEC in response to TNF-α stimulation, and interestingly, induction of CXCL10 was not observed in STING^–/–^ MHEC supernatants (Figure 4G). Moreover, antibody neutralization of CXCL10 in WT MHEC significantly decreased Th1 cell accumulation (Figure 4H) and almost completely inhibited TEM (Figure 4I). Taken together, these data demonstrate that STING in EC plays a critical role in T cell TEM across TNF-α–activated EC and identify the decreased levels of CXCL10 resulting from EC-STING deficiency as a plausible mechanism involved.

### T cell TEM is dependent on JAK/STAT and IFNI/IFNAR signaling.

**Figure 5:**
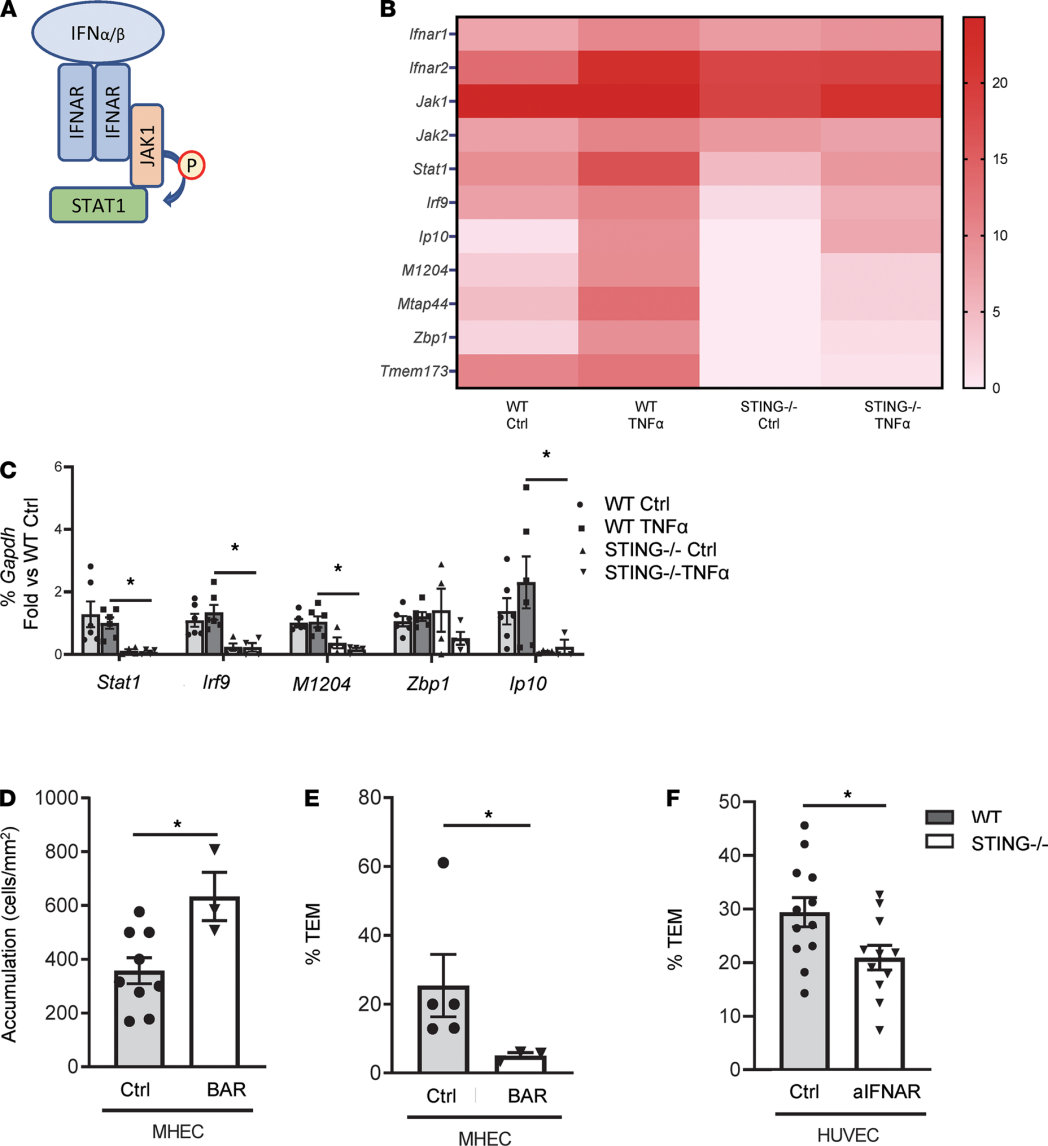
T cell TEM is dependent of EC JAK/STAT and IFNI. (**A**) Schematic of IFN-α and IFN-β molecules binding to the heterodimeric IFNAR and signal through JAK1 to recruit STAT1. (**B**) Representative heatmap corresponding to log_2_ values from RNA-seq of WT and STING^–/–^ control and 4-h TNF-α–stimulated MHEC (*n* = 3 mice/group were pulled for RNA-seq). (**C**) qPCR validation of STAT1 and the indicated ISG in WT and STING^–/–^ control and 4-h TNF-α–stimulated MHEC (*n* = 3 independent MHEC preparations, *n* = 2 replicates per condition). (**D**) Quantification of WT Th1 T cell accumulation. (**E**) WT Th1 T cell %TEM across 4-h TNF-α–stimulated MHEC treated with JAK1 inhibitor BAR. *n* = 3 independent experiments (duplicate coverslips for control). (**F**) Quantification of human CD3^+^ T cell %TEM across 4-h TNF-α–stimulated HUVEC with IFNAR blockade. *n* = 4 independent experiments, triplicate coverslips. Data are mean ± SEM. *P < 0.05; *t* test.

STING activation can lead to IFNI and ISG transcription (18), as well as to NF-κB activation (30). Our results indicate that 2 NF-κB–regulated molecules in response to TNF-α, ICAM1 and VCAM1, are not altered by the absence of STING. In contrast, CXCL10, an ISG downstream from IFNI, required for T cell TEM, is significantly decreased in STING^–/–^ MHEC. Thus, we hypothesized that STING modulation of TEM was through IFNI signaling. All IFNI molecules bind to the heterodimeric IFNAR and signal through JAK1, which recruits and activates STAT1 (Figure 5A), as well as induces the transcription and translation of ISG (31). A heatmap of RNA sequence (RNA-seq) analysis of WT and STING^–/–^ MHEC showed specific patterns of expression in the IFNAR-JAK/STAT-ISG axis (Figure 5B). We validated the expression of several genes in this axis by quantitative PCR (qPCR) and found that, as expected, the ISG *Irf9*, *Ip10*, and *M1204* were significantly decreased in STING^–/–^ MHEC at baseline and were not induced by TNF-α. Z*bp1*, in contrast, was not changed, and the levels of *Mtap44* were undetectable. STAT1 was not induced by TNF-α in WT EC and was also decreased in the absence of STING (Figure 5C). These data confirm that STING regulates IFNI responses in EC. These results led us to next investigate whether MHEC IFNI contributed to T cell TEM in response to TNF-α. To that end, we inhibited the JAK/STAT pathway downstream of IFNAR with Baricitinib (BAR) in MHEC and found that BAR increased accumulation of Th1 cells to MHEC (Figure 5D) but significantly inhibited TEM (Figure 5E). Furthermore, neutralizing IFNI in TNF-α–treated HUVEC resulted in decreased T cell TEM as compared with control (Figure 5F). Taken together, these results demonstrate that EC IFNI signaling contributes to T cell TEM in response to TNF-α and suggest a mechanism for STING regulation of TEM.

### Exogenous paracrine IFNI signaling contributes to leukocyte recruitment in response to TNF-α–induced peritonitis and to T cell TEM in vitro.

**Figure 6:**
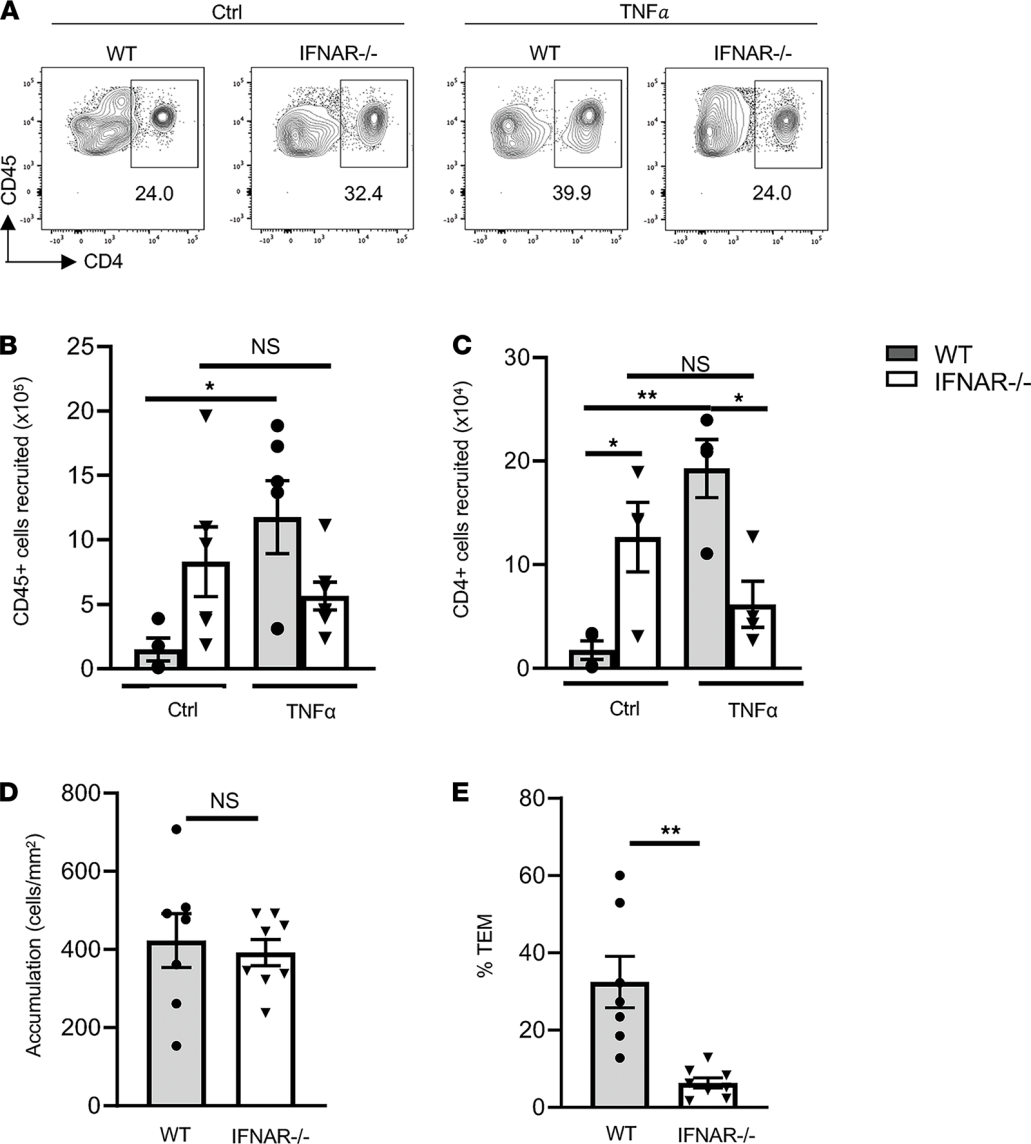
Exogenous Paracrine IFNI signaling contributes to leukocyte recruitment in response to TNF-α–induced peritonitis and TEM in vitro. (**A**–**C**) WT and IFNAR^–/–^ mice received PBS or TNF-α i.p., and the peritoneal lavage was harvested and analyzed by FACS. (**A**) Representative flow cytometric panels of CD45^+^CD4^+^ cells recruited to the peritoneal cavity. (**B** and **C**) Quantification of total CD45^+^ (**B**) and CD4^+^ (**C**) cells recruited cells to the peritoneal cavity (*n* = 4–7 mice/group). (**D** and **E**) WT Th1 cells were perfused across TNF-α–stimulated MHEC monolayers, and adhesion (**D**) and WT %TEM (**E**) were quantified from *n* = 3 independent experiments using duplicate or triplicate coverslips. Data are mean ± SEM. *P < 0.05 and **P < 0.01; 1-way ANOVA (**B** and **C**) and *t* test (**D** and **E**).

We further investigated this mechanism using the TNF-α–induced peritonitis model in IFNAR^–/–^ mice (Figure 6A) that are unable to initiate exogenous paracrine IFNI signaling (32). We found that decreased numbers of CD45^+^ cells (Figure 6, A and B) and CD4^+^ cells (Figure 6C) were recruited into the peritoneal cavity compared with WT control mice in TNF-α–treated mice. Interestingly, we noticed that PBS-treated IFNAR^–/–^ mice had significantly more CD45^+^ cells and, subsequently, more CD4^+^ cells at baseline compared with WT mice (Figure 6, B and C). To assess whether the impaired recruitment to TNF-α in IFNAR^–/–^ mice was due to defective IFNI signaling in EC, we performed TEM under flow conditions in vitro in TNF-α–treated MHEC. We found that Th1 TEM across IFNAR^–/–^ MHEC was significantly impaired as compared with WT MHEC, and we did not observe any differences in Th1 cell accumulation (Figure 6, D and E). Taken together, our data demonstrate that exogenous paracrine IFNI signaling in EC, via the IFNAR, contributes to leukocyte recruitment and T cell TEM in response to TNF-α in vivo and in vitro.

## Discussion

**Figure 7:**
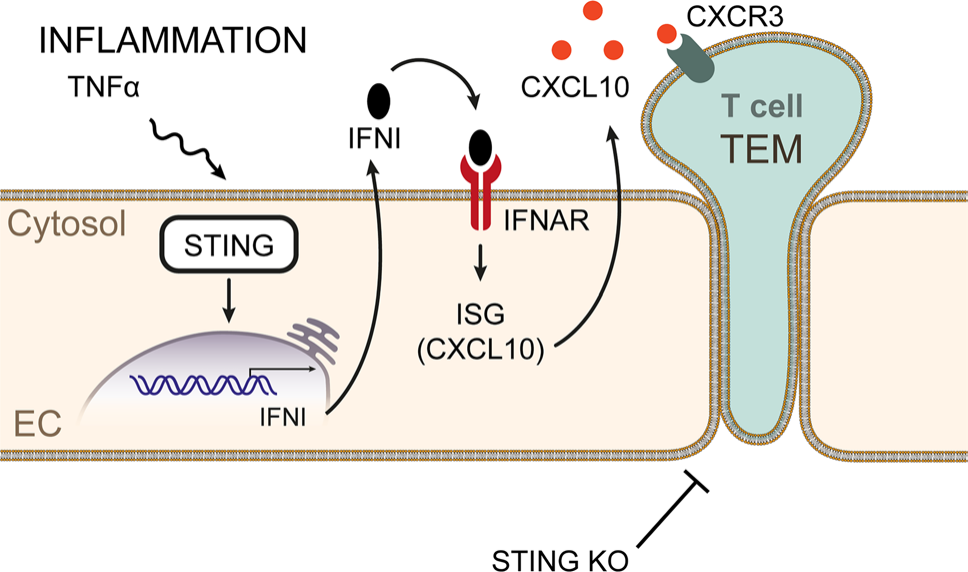
Schematic representation of the role of endothelial cell STING1 in T cell TEM and recruitment to sites of TNF-α–mediated inflammation. The absence of endothelial STING impairs TEM in a IFNI manner via the IFNAR and impairment of the IFNI induced gene CXCL10, a chemoattractant for T cells.

In the present study, we report a central role of EC-STING in T cell TEM. We demonstrate that global STING^–/–^ mice have impaired T cell recruitment in response to a TNF-α–induced model of peritonitis compared with WT mice. We also found that STING deficiency in EC, but not in T cells, results in impaired TNF-α–induced T cell TEM and that loss of STING has no effect on T cell adhesion. The specific role for STING in TNF-α–induced T cell recruitment was further corroborated in our newly generated EC-STING^–/–^ mice. Mechanistically, we found that STING^–/–^ EC had decreased expression of the ISG CXCL10 after TNF-α activation and that blocking paracrine IFNI signaling in EC similarly inhibited T cell TEM in vitro and T cell recruitment in vivo (Figure 7). Given the role of IFNI in antiviral and proinflammatory responses, most of our knowledge in the IFNI mechanisms induced upon STING function come from innate and adaptive immune cells (4, 18). Thus, our study identifies a central role for EC-STING and IFNI in T cell TEM, and it significantly advances our understanding of the role STING plays in the vasculature in response to sterile inflammation.

We selected TNF-α as a proinflammatory stimulus, common to diverse inflammatory settings, to induce peritonitis in mice, and we found that T cells were abundantly recruited by 24 hours in WT mice, as previously reported (19, 20), but were strikingly reduced in global STING^–/–^ mice. Notably, when STING^–/–^ mice were challenged with thioglycollate, we did not find significant differences between WT and STING^–/–^ mice in recruitment of myeloid cells. Consistent with no defect in myeloid cell recruitment, previous studies of peritoneal macrophages from STING^–/–^ mice have not reported differences in yields between WT and STING^–/–^ mice. Moreover, 3 days of thioglycollate peritonitis do not induce local increases in TNF-α (33), in line with our data showing no induction of T cell recruitment and no differences in T cell numbers between WT and STING^–/–^ mice in this model; this is in contrast to the TNF-α peritonitis model of inflammation. Our mechanistic studies using the combinations of STING^–/–^ T cells and STING^–/–^ MHEC, human primary T cells, and control and STING KD in HUVEC — combined with our results in EC-STING^–/–^ mice — further support a specific role for EC-STING in T cell recruitment and TEM in response to TNF-α.

We further teased out the mechanisms downstream of STING that participated in T cell TEM, and we investigated the NF-κB– and IRF3-dependent genes, such as ICAM1 and VCAM1 (NF-κB–dependent genes), and IFNI and ISG (IRF3-dependent genes) (34). We used a well-documented model system of T cell adhesion and TEM to investigate the role of EC-STING and these 2 downstream pathways (20, 23). Somewhat surprisingly, we found that TNF-α–activated STING^–/–^ EC expressed similar levels of ICAM1 and VCAM1 compared with their WT counterparts, given that these molecules are not only regulated by NF-κB in response to TNF-α but also play a central role in T cell adhesion and TEM (1). The expression of PECAM-1, which was not altered by TNF-α but was necessary for leukocyte TEM (35), also was not affected by deletion of STING. We and others have previously reported differential expression of ICAM1 and VCAM1 in mouse and human EC, with higher constitutive expression in MHEC as compared with HUVEC, and — in both cell types — further upregulation by TNF-α stimulation (36). Our data demonstrate that STING deficiency did not alter inducible adhesion molecule expression in either human or mouse EC models, and they support our conclusion that regulation of TEM by STING did not involve these classic components of TEM. In contrast, the ISG chemokine CXCL10 was significantly decreased in TNF-α–treated STING^–/–^ MHEC. Its receptor, CXCR3, is highly expressed on Th1 cells and is regulated by T-bet, the Th1 cell signature transcription factor (37). Rapid engagement of CXCR3 by CXCL10 and CXCL9 results in T cell adhesion to and TEM of EC monolayers, as we and others have previously reported (26, 27, 29). CXCL9 was below detectable levels in all conditions tested (not shown). Our results support the conclusion that STING regulates IFNI signaling required for CXCL10 production that promotes T cell TEM. In line with these data, we did not observe defective neutrophil recruitment or TEM in the absence of STING, supporting the conclusion that CXL10, which is not required for neutrophil recruitment, is specifically modulated by STING in EC and is required for T cell TEM (Figure 4). Interestingly, IFNI have therapeutic benefits in certain autoimmune inflammatory diseases and have been shown to prevent effector T cell migration to sites of inflammation through downregulation of CXCR3 (38).

STING activation leads to IFNI induction, which in turn signals through IFNAR to activate downstream JAK/STAT, resulting in transcription of several ISG in a well-described signaling feedback loop, as previously reported in myeloid cells (8). How this plays out in TEM regulated by STING remains to be investigated; however, our RNA-seq analysis of MHEC from WT and STING^–/–^ MHEC provides some insight. Our data indicate that STING does not regulate the expression levels of IFNAR or its downstream adaptor molecules STAT1 and STAT2. However, STING does significantly regulate the expression of several other ISG, in addition to CXCL10 at baseline, and also in response to TNF-α. Interestingly, in STING^–/–^ MHEC, not all ISG are equally inhibited upon TNF-α stimulation, suggesting that TNF-α interferes with the IFNI/IFNAR signaling feedback loop regulated by STING. Our data using the JAK/STAT inhibitors, IFNAR blockade, or IFNAR^–/–^ EC supported a critical role for IFNI downstream of STING in triggering T cell TEM. These data are in line with reports showing that IFNI signaling and STING activation in EC enhance vascular inflammation (12), with the suppression of inflammation in vasculitis by JAK/STAT inhibitors (39). Our findings demonstrating that IFNAR^–/–^ mice have decreased T cell recruitment in TNF-α–induced peritonitis further support this mechanism. Our results also reveal that significantly more leukocytes were present in the peritoneal cavity of IFNAR^–/–^ at baseline, and this could be indicative of either a homing defect or defective tonic IFNI signaling. This tonic signal, reported to be central to immune cell homeostasis (40, 41), may likely be critical for vascular homeostasis by preventing leukocyte infiltration into tissues in resting conditions.

Our data provide, to the best of our knowledge, the first experimental evidence that EC-STING is central to CD4^+^ T cell TEM using potentially novel mechanisms that do not involve downregulation of the transcriptionally regulated EC classic adhesion molecules involved in TEM. We suggest that STING regulation of TEM in response to TNF-α is predominantly biased toward the IFNI signaling branch and less so through NF-κB, and that the decreased levels of CXCL10 in STING^–/–^ EC is the likely explanation for impaired T cell TEM. However, while STING deficiency in EC inhibits TEM but not the preceding adhesion step, CXCL10 neutralization inhibits both steps, suggesting that an additional mechanism modulated by STING may be required for TEM. Moreover, how TNF-α activates STING to induce IFNI in EC remains to be defined. The CXCL10 promoter has IFN-stimulated response elements (ISRE), which are bound by IFN regulatory factors (IRF). Thus, it is likely that TNF-α activation of STING transcriptionally regulates CXCL10 gene expression through IFNI-dependent mechanisms (42). The CXCL10 promoter also contains NF-κB binding sites, and given that TNF-α induces NF-κB, CXCL10 could also be activated this way. Additional studies also are needed to pinpoint the EC molecular signals in diapedesis that are dependent upon STING. One possibility is that STING deficiency alters calcium signaling through modulation of TRIPC channels, which were recently shown to contribute to leukocyte diapedesis (43). In addition, our RNA-seq analysis and subsequent validation of genes in the IFNAR/ISG axis identified differences between WT and STING^–/–^ EC that are unrelated to TEM and may involve other functions of EC-STING beyond leukocyte TEM and inflammation, which are beyond the scope of these studies. Based on these findings, future studies using the newly generated Cad5^ERTCre2+/–^ STING^fl/fl^ may focus on how EC-STING regulates other aspects of endothelial function, such as blood pressure regulation or responses to EC injury. Lastly, while we define a central role for STING in T cell recruitment, how TNF-α and STING drive sustained inflammation in EC in a chronic setting has not been investigated here. CXCL10, for instance, is critical in leukocyte recruitment that occurs in heart failure (29), and IFNI signals downstream of STING have been reported to fuel cardiac damage in response to myocardial ischemia (14).

In conclusion, our study supports a potentially novel role for EC-STING in T cell TEM and provides mechanistic insight into how to modulate STING or its downstream IFNI pathway to control undesired T cell tissue and organ infiltration during inflammation.

## Methods

### Reagents.

Recombinant hTNF-α (catalog 300-01A) and mTNF-α (catalog 315-01A), and rmIL12 (catalog 210-12), rmIL2 (catalog 212-12), carrier-free hSDF-1α (catalog 300-28A), and mSDF-1α (catalog 250-20A) were purchased from Peprotech. Thioglycollate broth (REMEL, R064702) was purchased from Thermo Fisher Scientific. Recombinant mouse E-selectin (catalog 575-ES-100), P-selectin (catalog 737-PS-050), ICAM1 (catalog 796-IC-050), VCAM1 Fc-chimeras (catalog 643-VM-050), and mouse CXCL10/IP-10/CRG-2 antibody (AF-466-NA) were purchased from R&D Systems. Antibodies to mouse cytokines and adhesion molecules were as follows: anti-CD3 (clone 145-2C11, catalog 100253), anti-CD28 (clone 37.51, catalog 102102), anti-IL4 (catalog 504102), LFA-1-PE (catalog 1053103), VLA-4-FITC (catalog 103605 ), PECAM-1-FITC (catalog 102506 ), ICAM1-FITC (catalog 116105), and VCAM1-PE (catalog 105714), as well as immune cell marker–conjugated fluorophores CD45-BV711 (catalog 109847), CD45-PE (catalog 109808), CD3-APC/Cy7 (catalog 100330), CD4-FITC (catalog 100406), CD11b-PerCP (catalog 101228), GR1-APC (catalog 108412), and CD11c-PE (catalog117308), were purchased from BioLegend. Antibodies to human ICAM1 (Hu5/3) and VCAM1 (E1/6) have been reported previously (44), and PECAM-1 (P2B1, Developmental Studies Hybridoma Bank) were used as hybridoma conditioned media for flow cytometry studies. BAR (catalog 16707) was purchased from Cayman Chemical. IFNAR blockade (MAR1-5A3) was purchased from BD Pharminogen. Tamoxifen (catalog T5648) and 4-Hydroxytamoxifen (catalog 94873) were purchased from MilliporeSigma. STING/TMEM173 (catalog 13647) and β-actin antibodies (catalog 4967) were purchased from Cell Signaling Technologies. RosetteSep Human T Cell Enrichment Cocktail (catalog 15021) was purchased from Stemcell Technologies.

### Mice.

C57BL/6 mice were purchased from the Jackson Laboratory. STING^–/–^ mice were donated by Alexander Poltorak (Tufts University) (24). IFNAR^–/–^ and STING^fl/fl^ mice were provided by Shruti Sharma (Tufts University). Cdh5^CreERT2+/–^STING^fl/fl^ mice were generated by crossing mice expressing vascular cadherin promoter–driven inducible Cre (Cdh5^CreERT2+/–^), donated by Hong Chen (Boston Children’s Hospital, Boston, Massachusetts, USA) (45), with STING^fl/fl^ mice in order to generate the EC-STING mice and EC-STING^–/–^ mice after tamoxifen administration. All mice were bred and housed in the Tufts University School facilities.

Mice were sacrificed at 8–12 weeks of age for harvest of naive T cells or used between 8 and 12 weeks of age for peritonitis experiments and for tissue and blood harvest. The efficiency of genomic recombination after tamoxifen treatment in mice was determined by PCR analysis of DNA isolated from the heart, splenocytes, purified EC, and T cells using primers P1 5′→3′ ACACGCTCTGTTTACTATGAACCTC, P2 5′→3′GGGGGAAGGAGAGAACTGAC. To determine the efficiency of cell-specific STING KD after Cre recombinase, CD31^+^ and CD4^+^ cells were isolated from 3 Cdh5^CreERT2+/–^ STING^fl/fl^. CD31^+^ cells were grown in 12-well plates, and CD4^+^ cells were grown in 24-well plates for 3 days and were further cultured in presence or absence of 4 hydroxy TMX (10 μmol/L; MilliporeSigma, catalog 94873) for 24 hours. Cells were washed and cultured for an extra 24 hours. Treated cells from each well were lysed in 50 μL of loading buffer, and 20 μL were loaded to run a Western blot.

### In vivo TNF-α– and thioglycollate-induced peritonitis models of leukocyte recruitment.

Eight- to 12-week-old age-matched WT, STING^–/–^, IFNAR^–/–^, and EC-STING female mice were injected i.p. with either PBS or PBS containing TNF-α (100 ng/mouse) (20), and 4 hours and 24 hours later, cells recruited to the peritoneal cavity were harvested, counted, and stained for the indicated immune cell markers and analyzed by flow cytometry. For peritonitis experiments using EC-STING mice, mice were treated for 3 consecutive days with i.p. injections of tamoxifen (75 mg/kg dissolved in sunflower oil + 10% ethanol) followed by 2 days of rest prior to TNF-α stimulation. In thioglycollate-induced peritonitis, 8- to 12-week-old age-matched WT and STING^–/–^ mice were injected i.p. with PBS or thioglycollate (3 mL of 3% aged thioglycollate broth), and 72 hours later, cells were harvested by peritoneal lavage, counted, stained for the indicated immune cell markers, and analyzed by flow cytometry.

### Preparation of effector T cells and mouse neutrophil isolation.

Naive CD4^+^ T cells were isolated from spleen cell suspensions of WT or STING^–/–^ mice by positive selection using magnetic beads (Miltenyi Biotec). Isolated CD4^+^ T cells were differentiated into Th1 T cells as described (22) by stimulation with anti-CD3 (5 μg/mL) and anti-CD28 (1 μg/mL) in the presence of IL-12 (10 ng/mL), IL-2 (25 U/mL), and anti–IL-4 (50 ng/mL). Three days following stimulation, Th1 cultures were split 1:1 with medium containing IL-2 (25 U/mL). Differentiated T cells were harvested on day 4, counted, resuspended in fresh medium, and immediately used for experiments. Neutrophils were isolated from BM suspensions by negative selection using the EasySep kit (Stemcell Technologies) (46).

### Evaluation of T cell adhesion and TEM under defined flow conditions in vitro.

MHEC were generated from WT and STING^–/–^ mice and grown to confluence as previously described (47). Briefly, T cell adhesion and TEM were determined under defined laminar flow conditions in a parallel plate apparatus using video microscopy (total original magnification, ×200) and the Nikon Elements NIS software as previously described (36). Briefly, MHEC monolayers were treated with TNF-α (125 ng/mL) for 4 hours and with stromal cell–derived factor-1α (SDF-1α) (250 ng/100 μL) for 15 minutes prior to perfusing 2 × 10^6^ Th1 cells in the flow chamber. MHEC were treated with 10 μM BAR, a JAK1/2 inhibitor, 20 minutes prior to Th1 perfusion. MHEC were cultured with CXCL10 blocking antibody (0.5 μg/mL) for 4 hours prior to Th1 perfusion. Percent TEM is represented as (TEM cells/total accumulated) × 100. T cell interactions with immobilized ICAM1 (20 μg/mL) and VCAM1 (40 μg/mL) were evaluated using the same videomicroscopy system determining accumulated cells after perfusion of PMA-treated T cells (50 ng/mL, 5 minutes, to induce integrin activation) at a concentration of 1 × 10^6^ cells/mL at an estimated shear stress of 1 dyne/cm^2^ (ICAM1) and 0.5 dynes/cm^2^ (VCAM1). Adherent cells were quantified in 5–6 different fields of view. In studies involving mouse neutrophils, neutrophils were isolated from the BM extracted from the femur and tibias of WT mice using an EasySep kit from Stemcell Technologies according to the manufacturer’s instructions (46). Neutrophil purity was > 90% as determined by flow cytometry for CD11b/Ly6G double-positive cells.

### STING KD in HUVEC and TEM assays.

The Broad Institute Genetic Perturbation Platform (GPP) single-guide RNA (sgRNA) Designer was used to select CRISPR guide sequences. The human STING-targeting lentivirus was generated using the vector LentiCRISPR v2 (a gift from Feng Zhang, Broad Institute, Cambridge, Massachusetts, USA; Addgene plasmid 52961) expressing the guide sequence 5′-GCTGGGACTGCTGTTAAACG-3′. HUVEC (sc-2) were transduced with lentivirus and, 24 hours later, selected with 0.5 μg/mL puromycin in culture media for 5–6 days. Cells were then plated on fibronectin-coated glass coverslips 2 days prior to flow assays. Human CD3^+^ T cells (>95% purity) were isolated by negative selection (Stemcell Technologies) from anticoagulated whole blood obtained from healthy volunteers. Blood was obtained from volunteer donors according to Brigham and Women’s Hospital IRB–approved protocols for protection of human subjects, and all volunteer subjects gave informed consent, in accordance with the Declaration of Helsinki. Isolated T cells were cultured overnight in RPMI containing 10% FCS, 1% glutamax (Invitrogen, catalog 35050061), 1% penicillin/streptomycin (Invitrogen, catalog 15140122), and 10 ng/mL IL-2 (Peprotech). TEM assays were performed on HUVEC monolayers treated for 4 hours with 10 ng/mL TNF-α and with 50 ng/mL SDF-1α for 20 minutes prior to the start of the assay. A bolus of 2 × 10^6^ T cells at a concentration of 1 × 10^7^ T cells/mL was drawn across the monolayers in a parallel-plate flow chamber maintained at 37°C and allowed to adhere. Then, a single field of view was recorded using MetaMorph software for 10 minutes at a shear of 1.5 dynes/cm^2^. In studies involving neutrophils, human polymorphonuclear cells (PMNs; >95% pure) were isolated from whole blood drawn from healthy volunteers, kept at 8°C, and used immediately as described (48).

### Flow cytometry.

Flow cytometry was performed to corroborate the differentiation of Th1 cells as described, the expression levels of VCAM1 and ICAM1 ligands on Th1 cells, and the recruitment of immune cells into the peritoneal cavity in vivo using the antibodies listed in the Reagents section. The data were acquired on a FACS LSRII flow cytometer and analyzed using FlowJo software.

### Western blots.

WT STING^–/–^ cells were washed in cold PBS, lysed in RIPA buffer with protease inhibitors. Protein concentrations were determined with the BCA kit (Thermo Fisher Scientific). About 50ug of lysate were run in an SDS-PAGE gel. The blots were incubated with STING/TMEM173 antibody (1:500; Cell Signaling Technologies, catalog 13647) or β-actin antibody (1:5000; Cell Signaling Technologies, catalog4967). In the experiments involving Cdh5^CreERT2+/–^ STING^fl/fl^ mice, MHEC were cultured in 12-well plates and treated with 10 μmol/L of 4OH-TMX or vehicle for 24 hours. Cells were lysed directly in 2× laemmli sample buffer (Bio-Rad).

### RNA-seq.

Total RNA was isolated from unstimulated and TNF-α–stimulated (125 ng/mL) MHEC from WT and STING^–/–^ mice using TRIzol. A directional cDNA library using TrueSeq kit was prepared and sequenced using MiSeq (Illumina). The data were analyzed using the Tuxedo tools (Bowtie, TopHat, Cufflinks/Cuffdiff/Cuffmerge/CuffCompare, and CummbeRbund). The resulted fastq files were first mapped to the mouse reference genome (NCBI/mm9) using TopHat (V2.0.0). The normalization, quantification, and different expression analysis of transcript level was determined using Cufflinks and Cuffdiff. The data were then visualized using Integrated Genome Viewer (from BROAD Institution), as well as CummbeRbund. Raw and processed sequencing data have been deposited in Gene Expression Omnibus (GEO) repository under accession no. GSE178200 (https://www.ncbi.nlm.nih.gov/geo/query/acc.cgi?acc=GSE178200).

### CXCL10 quantification.

CXCL10 was evaluated in supernatants of WT and STING^–/–^ MHEC treated with TNF-α (125 μg/mL, 4 hours) using Eve Technologies Corporation Discovery assays. 

### Statistics.

Data were analyzed using GraphPad Prism software and presented as the mean ± SEM. Statistical analyses were done by Mann-Whitney *U* nonparametric unpaired or 2-tailed Student *t* test when comparing 2 groups. Multiple-group comparisons were performed by 1-way ANOVA with Bonferroni post hoc test where indicated. Differences were considered statistically significant at *P* ≤ 0.05.

### Study approval.

All mice used were bred in accordance with the guidelines of the committee of Animal research at Tufts University School of Medicine, Tufts Medical Center, and the NIH Animal research guidelines. All animal studies were approved by the Tufts University IACUC. Blood was obtained from volunteer donors according to Brigham and Women’s Hospital IRB–approved protocols for protection of human subjects, and all volunteer subjects gave informed consent, in accordance with the Declaration of Helsinki

## Author contributions

MA performed all the in vivo and the in vitro experiments involving mouse EC, analyzed the data, and drafted the manuscript. GAN performed all the experiments involving HUVEC. KK, FJCS, and SAS participated in the experiments involving EC-STING^–/–^ mice characterization and 4-hour peritonitis experiments, and in the generation of MHEC. ALB and SAS performed qPCR studies to validate RNA-seq genes. VI and AP performed the RNA-seq study, and SS provided intellectual input and ideas for the IFNAR studies. FWL oversaw and designed the studies in HUVEC and provided intellectual feedback in the manuscript writing, and PA designed the experiments and wrote the manuscript. All authors participated in revising the writing of the manuscript and intellectually contributed. Co–senior author order was agreed upon, with PA noted as the last author for leading the manuscript writing.

## Supplementary Material

Supplemental data

## Version 1 06/22/2021

In-Press Preview

## Version 2 08/09/2021

Electronic publication
