# Supplementary material for: Endothelial STING controls T cell transmigration in an IFNI-dependent manner
Source: JCI Insight. 2021 Aug 9;6(15):e149346. doi: 10.1172/jci.insight.149346 (PMC8410041; doi:10.1172/jci.insight.149346)
Supplement: Supplemental data [file jciinsight-6-149346-s273.pdf]

Supplemental Figure 1

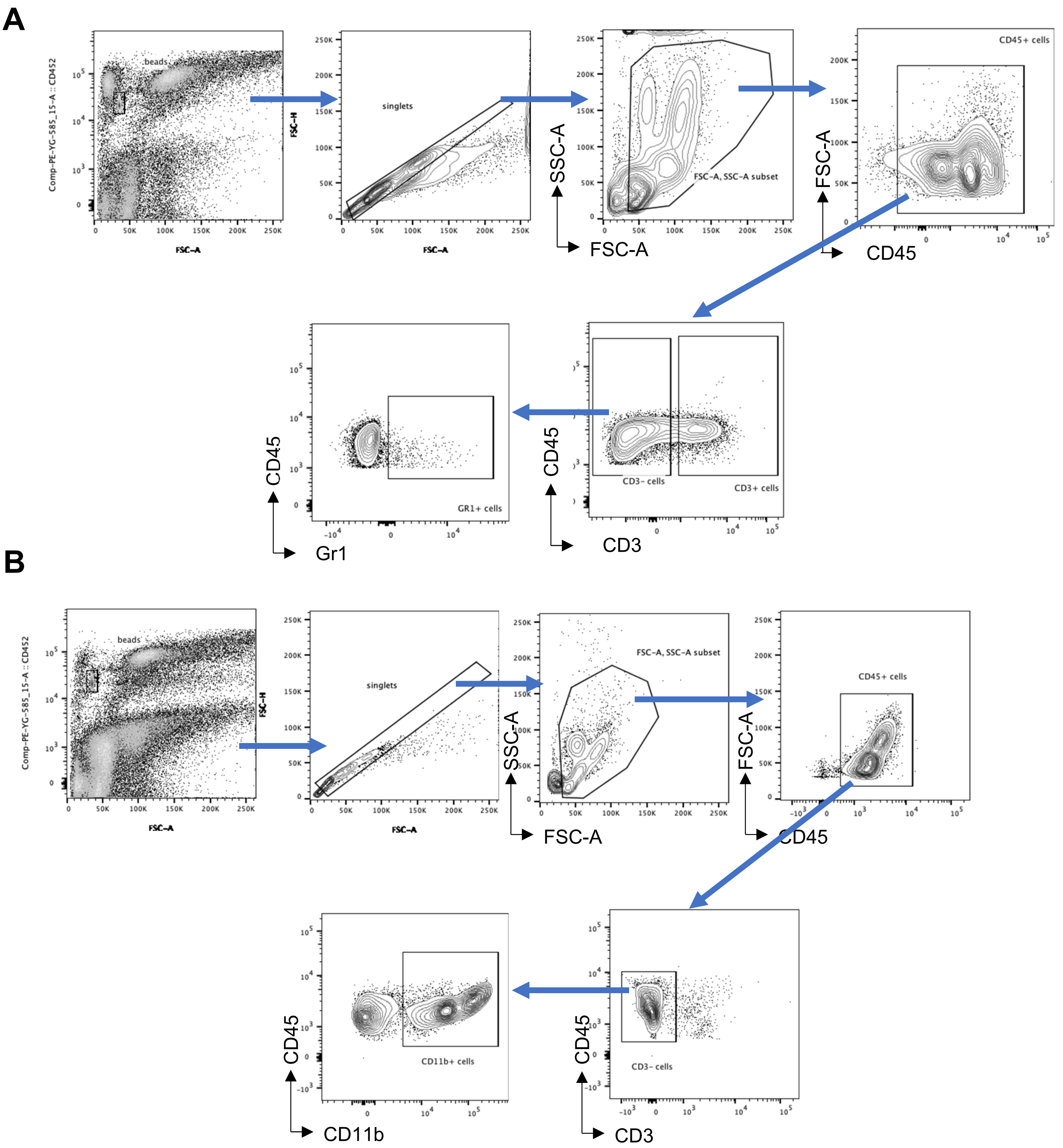

Supplemental Figure 1

Representative gating strategy for the *in vi*vo experiments of peritonitis. Plots represent the peritoneal lavage of WT mice treated with TNF-α 24h **(A)** and with Thioglycollate 3 days **(B).**

Supplemental Figure 2

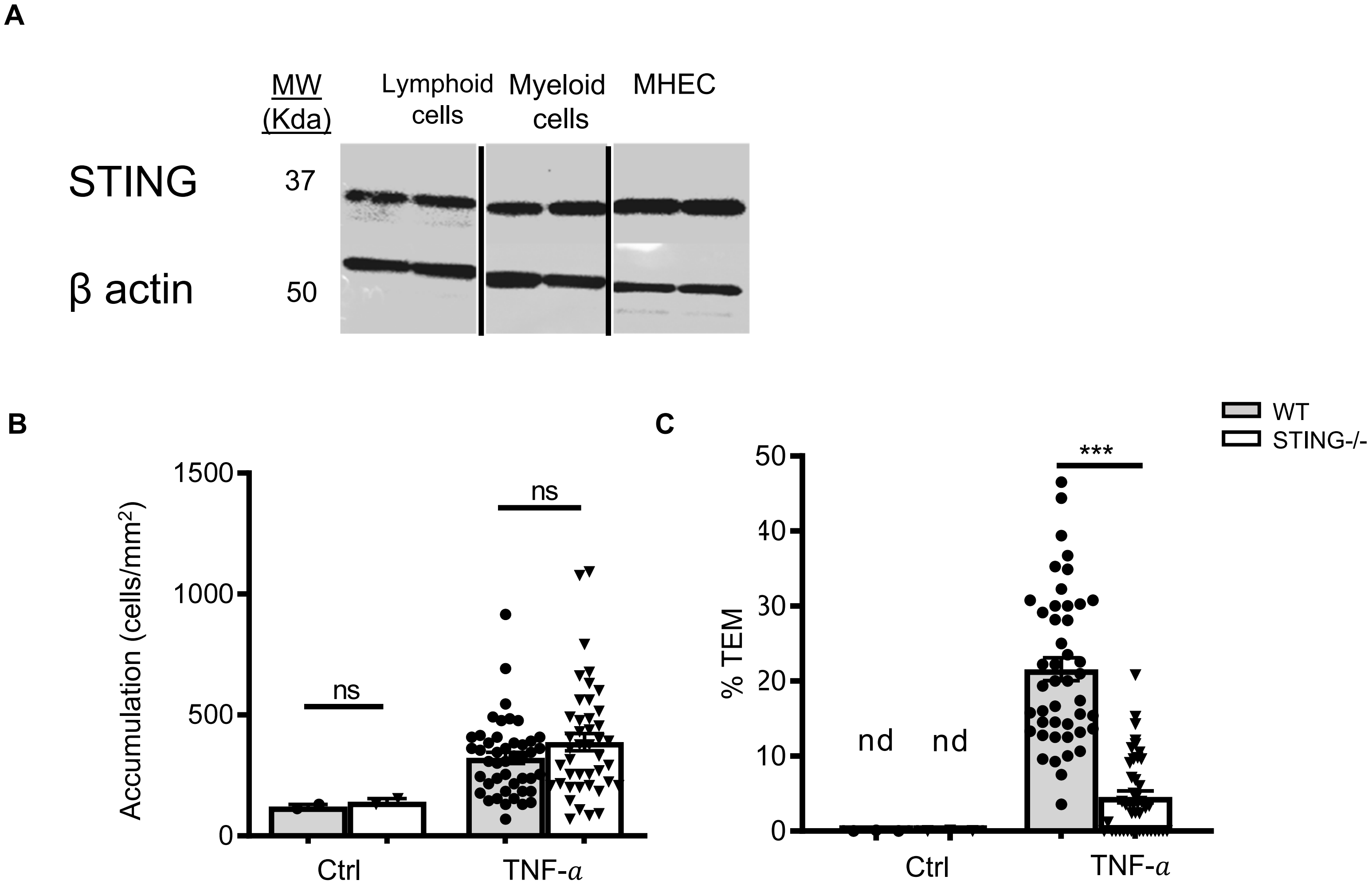

Supplemental Figure 2

Cultured MHEC, myeloid cells and column-isolated CD4+ T cells from WT mice were lysed and analyzed by immunoblot to evaluate STING expression and β-actin as a loading control. (**A)** One representative blot is shown of 3 independent experiments; each lane is an independent cell preparation. **(B-C)** Quantification of accumulation and %TEM respectively of WT Th1 cells perfused across unstimulated and TNF-α-stimulated WT and STING-/- MHEC. n=6 independent experiments (TNF-α-), and n=2 (non stimulated Ctrl), in duplicate or triplicate coverslips. 1-way ANOVA **(B-C).**

Supplemental Figure 3

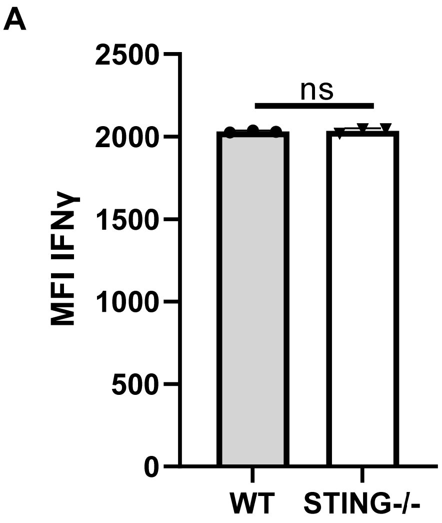

Supplemental Figure 3

(A) WT and STING-/- Th1 cells were differentiated *in vitro* as described in methods, permeabilized and stained for intracellular IFN-γ. Quantification of the median fluorescent intensity of IFN-γ per cell is represented. n=3 independent T cell preparations/group. t-test.

Supplemental Figure 4

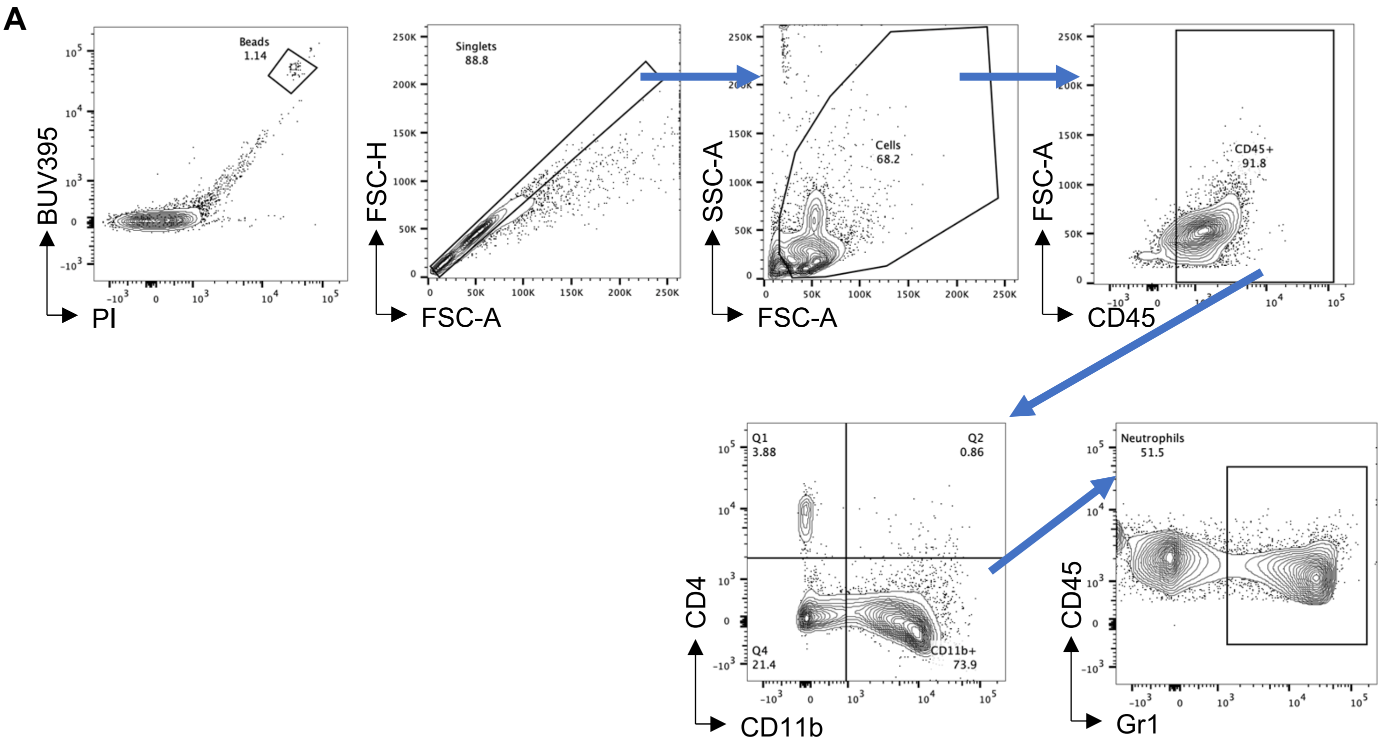

Supplemental Figure 4

(A) Representative gating strategy for the *in vivo* experiments of 4h TNF-α induced peritonitis. Plots represent the peritoneal lavage of Cad5 $^{ERTCre2+/-}$STING$^{fl/fl}$ treated with tamoxifen or vehicle control 4 hours after TNF-α injection. The top left panel gate represents the counting beads.

Supplemental Figure 5

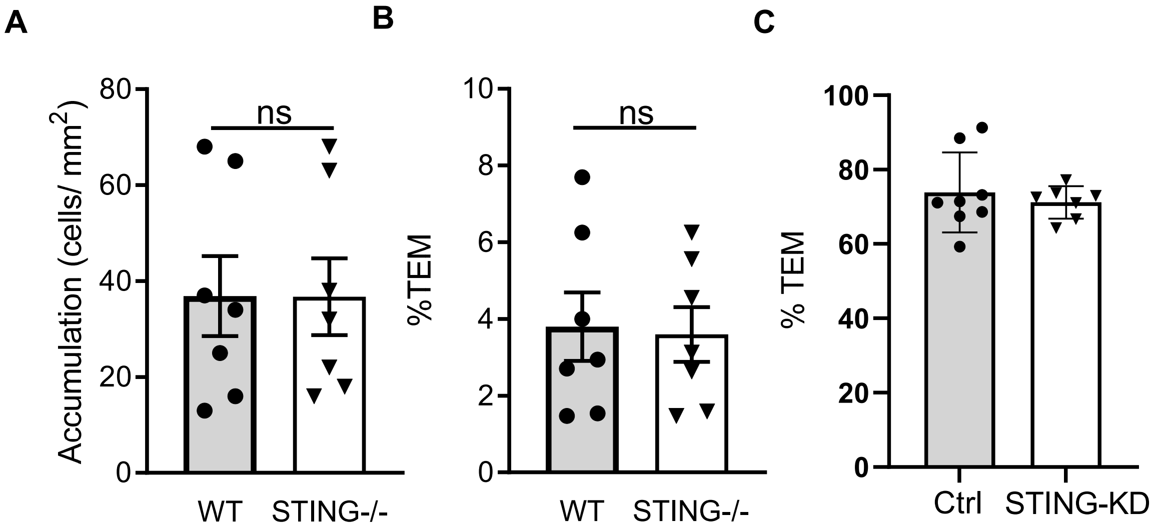

Supplemental Figure 5. STING deficiency does not result in decreased neutrophil TEM across mouse and human primary endothelial cells. (**A** and **B)** Mouse bone marrow derived neutrophils were perfused across cultured MHEC from WT and STING-/- mice treated 4h with TNF-α, and quantification of accumulation **(A)** and %TEM **(B)** was determined in n= 3 independent experiments, in duplicate coverslips. **(C)** Neutrophils isolated from human blood were perfused under flow conditions across WT and STING KD HUVEC stimulated for 4h with TNF-α and %TEM was quantified. n=8 (Ctrl) and n=7 (STING-KD) coverslips from n=2 independent experiments.
